# How to review a surgical paper: a guide for junior referees

**DOI:** 10.1186/s12916-016-0578-6

**Published:** 2016-02-14

**Authors:** Philip F. Stahel, Ernest E. Moore

**Affiliations:** Department of Orthopedics and Department of Neurosurgery, University of Colorado, School of Medicine, Denver Health Medical Center, 777 Bannock Street, Denver, CO 80204 USA; Department of Surgery, University of Colorado, School of Medicine, Denver Health Medical Center, Denver, CO USA

**Keywords:** Evidence-based medicine, Qualified referee, Peer-review process, Publication bias

## Abstract

Reviewing a surgical manuscript is not an easy task, and there is no formal training available for young referees in the early stage of their careers. Accepting a peer review assignment represents a personal honor for the invited referee and a fundamental ethical responsibility towards the scientific community. Designated reviewers must be accomplished and knowledgeable in the area of the respective topic of investigation. More importantly, they must be aware and cognizant about the cardinal ethical responsibility and stewardship for ensuring the preservation of scientific knowledge of unbiased and unquestionable accuracy in the published literature. Accepting a review assignment should never be taken lightly or considered a simple task, regardless of the reviewer’s level of seniority and expertise. Indeed, there are multiple challenges, difficulties, and ‘hidden dangers’ that jeopardize the completion of a high-quality review, particularly in the hands of less experienced or novice reviewers. The present article was designed to provide a brief, concise, and practical guide on how to review manuscripts for the ‘junior referee’ in the field of surgery.

## Introduction

When approached with a specific peer review request, refereeing candidates must first question their own qualifications and ability to perform a high-quality and unbiased evaluation [1]. In general, a suitable reviewer should be an active clinician-scientist who works in the same specialty and subspecialty of research that matches the topic and focus of the manuscript of interest. Why should a designated referee accept a reviewing task? Most clinicians, surgeon scientists, and basic researchers are confronted with daily tasks related to patient care, grant writing, and other competing project deadlines. New review requests are therefore typically considered an unnecessary and distracting burden, and the temptation of rejecting or even ignoring a new assignment is understandable. However, there are compelling ‘hidden incentives’ for referees to accept a new reviewing assignment, including (1) the possibility to be on the cutting edge of science and learn what is new in a specific field (new questions, concepts, and new surgical techniques); (2) the opportunity to mentor other authors in the field with encouragement and inspiration by helping to improve the quality of their work; and (3) the ability to contribute to the quality of evidence-based practice in a specific surgical discipline. Of note, it is considered a professional courtesy for commissioned referees to either accept or decline a review request as soon as possible, and to submit their evaluations before expiration of the respective deadline, which is currently set at 2 weeks for most journals.

## Fundamentals for reviewing a surgical manuscript

The standard requirements of how to perform a quality peer-review for general biomedical journals are covered elsewhere and are beyond the scope of this article. In the field of surgery, referees should be aware of selected nuances and distinct intricacies when confronted with the task of assessing a surgical manuscript [2]. For this purpose, there are a number of high quality articles in the published literature that provide excellent guidance to the ‘surgeon referee’ in specific arenas of peer review, including surgical outcomes research, evaluation of meta-analyses, randomized controlled trials, statistical analysis, and how to assess power and sample size [3–9]. Furthermore, most peer-reviewed biomedical journals have endorsed uniform standardized reporting guidelines for clinical trials, randomized studies, case reports, and meta-analyses of the published literature, e.g. CONSORT, QUOROM, PRISMA, STARD, STROBE, TREND, etc. [10–18]. These standardized guidelines are available elsewhere as an important resource for peer reviewers and are therefore not part of the scope of the present article. Instead, we aim to provide a simple and pragmatic checklist approach for ‘junior referees’ who are confronted with the task of evaluating a surgical paper.

The reviewer should consider screening a new submission in a standardized fashion (introduction, hypothesis, methodology, outcome measures, interpretation of the data, validity, and relevance of the conclusions). The following checklist provides a standard guidance through the analytic aspects of the review process:

### Why was the study performed? (Introduction/Hypothesis)

The introduction should provide a compelling rationale for conducting the proposed study. Do the authors define a relevant knowledge gap? Have they given appropriate credit to previous work in the field? Is the hypothesis clinically relevant and of scientific merit? In other words, does the study address an important unresolved problem in the field of surgery? Will the answer to the study question contribute to improvement in the quality of the clinical care delivered to surgical patients, or help resolve a previously unknown basic experimental question? Perhaps the easiest method to assess the quality of the introduction is through implementation of ‘the known, the unknown (knowledge gap), and the objectives (hypothesis)’ framework [19].

The junior referee should be aware that many manuscripts submitted for publication lack a defined a priori hypothesis, which should immediately question the validity of the study. The ever increasing competitiveness in research, in conjunction with decreasing opportunities for grant funding, may incentivize researchers to fragment (or ‘salami-slice’) results from a single study into multiple papers or to duplicate or publish identical datasets redundantly. This is a problem of critical concern since redundant publications ‘dilute’ the pool of truly existing insights and contribute to publication bias and flawed conclusions in meta-analyses and clinical guidelines [20]. Thus, it is the referee’s duty to subject the manuscript to a ‘truth test’ question on why the authors performed the current study; is the paper submitted for their own academic merit (‘publish or perish!’) or truly intended to address an important research question? First and foremost, check the hypothesis and its relevance!

### How was the study performed? (Methodology)

What is the study design? As the study design will ultimately determine the level of evidence according to the established evidence-based medicine criteria, it is imperative for the referee to scrutinize the underlying study design and to clarify and correct the true nature of the design, if needed. Most surgical papers are reflective of either a prospective or retrospective cohort study. The referee must be cognizant that the main hallmark of a prospective cohort study is the fact that, at the time of study inception, none of the study subjects had yet developed any of the outcomes of interest; any study design that does not meet this requirement is retrospective by definition. Beware that many submitted papers allegedly report ‘prospective’ data that, on coherent scrutiny of the study design, are unmasked as retrospective observational studies. One classic example supportive of this notion is a ‘retrospective analysis of a prospective database’, which is, by definition, reflective of a retrospective study design as the outcomes had already occurred at the time the study was initiated. Interventional studies or clinical trials are, by definition, prospective in design. Their main distinguishing feature is based on the participants’ exposure determined by an experimental intervention assigned by the investigators, e.g. a medical treatment or new surgical technique. In contrast, prospective cohort studies are observational and not interventional. In randomized controlled trials (RCTs), the assignment of subjects to one of the comparative treatment groups is performed by random allocation in order to mitigate the influence of confounding factors. Of note, many submitted surgical papers that claim to be reflective of a randomized trial do not stand the test of true level 1 evidence. It is therefore the referee’s obligation to scrutinize RCTs according to the CONSORT guidelines [11]. Frequent flaws in alleged RCTs are absence of a clear disclosure of the concealed allocation modality and the lack of an intention-to-treat analysis of the data. Beware of selected submissions that claim to represent a ‘surgeon-randomized’ study design; this basically implies that patients have been allocated to distinct surgical procedures according to the individual surgeon’s expertise, but not by random allocation. Such a study design is reflective of patient selection per surgeon’s convenience and availability and should therefore not be labeled with the ‘randomized’ designation reserved for RCTs [21].

In surgery, many submitted papers are frequently based on large databases because of their public availability. Unfortunately, many of these repositories are for administrative purposes and consequently do not contain the elements essential to address the study hypothesis. For example, post-injury coagulopathy is a very dynamic process that demands accurate documentation at frequent, early time-points to address the impact of varying transfusion practices; simply quantifying the sum of blood products administered within the first 24 h after injury leads to a ‘survival bias’, implying that patients who succumb early from their injuries will not have lived long enough to receive more blood products [22]. Moreover, many clinical databases represent voluntarily submitted data that has not been validated. The National Trauma Data Bank is a notorious example of these limitations [23]. In addition, the confounding variable of observer variation must be taken into consideration when assessing the quality of the underlying study design in surgical trials. This entity reflects the variability in measurements obtained by two or more observers examining the same set of data (‘inter-observer variation’) or the variability of measurements by one single observer examining the same data more than once (‘intra-observer variation’). Finally, the methodology must provide unequivocal inclusion and exclusion criteria for patient enrollment and the results must match the numbers of patients stratified by those criteria in the methods section. A crucial aspect for defining patient cohorts is whether these were enrolled consecutively; non-consecutive enrollment implies introduction of selection bias, which limits the scientific validity and credibility of the study.

### Are the outcome measures and analytical methods appropriate?

The study design should define one single primary outcome measure used as the main variable to either confirm or reject the null hypothesis. Frequently used outcome measures in surgical trials include in-hospital mortality, length of hospital stay, ventilator-dependent days, surgical complications, and functional or radiographic outcome scores. The primary outcome parameter is used to calculate the statistical power (1-β) of the study. There can be multiple ancillary (secondary) outcome measures to support the main findings. The referee has to assess whether the selected variables of interest are suitable to test the hypothesis, and if confounding factors have been taken into consideration in the elimination of bias that may lead to flawed interpretation of the results. A common error is to present data as normatively distributed (mean ± standard error of the mean) rather than median ± interquartile range. Another important aspect to take into consideration is the appropriateness of the statistical analysis. Most submitted manuscripts report significant or even highly significant results that may remain questionable if adequately scrutinized from the reviewer’s vantage point. The main question to ask is whether the statistically significant data (*P* <0.05) is clinically relevant (type 1 error). As the magnitude of the *P* value depends on sample size, minimal differences between study groups can become statistically significant in sufficiently large sample sizes. The question is whether such negligible changes are truly clinically relevant (for example, the demonstrated improved life expectancy after a surgical procedure by just a few days, etc.). This problem is of increasing importance when analyzing studies that are based on large multicenter databases or national registries with thousands or even millions of patients enrolled. The analysis of such extensive databases will make the most minimum differences in outcome parameters appear statistically significant. It is the reviewer’s duty to determine if those data are truly clinically relevant. Reciprocally, underpowered studies may not establish statistical significance despite dramatic clinical implications, purely due to small cohort sizes (type 2 error). Therefore, it is imperative to ensure that an adequate a priori power analysis based on the primary outcome measure and able to either confirm or reject the null hypothesis has been performed. Many referees may not feel qualified to assess the quality of the statistical analysis in detail, and should therefore have a low threshold to request a formal statistical review by the journal’s dedicated professional biostatistician.

### Are the conclusions supported by the data shown?

The discussion section of the paper should, in essence, address the question: *“How does the article I read today change what I recommend to my patients tomorrow?”* [24]. The discussion should be introduced in such a manner that a relevant conclusion can be offered. In general, the discussion should follow a logical sequence, e.g. summary of main findings, comparison to other previous publications on the topic, discussion of alternative explanations for the observations, clinical relevance, limitations of the study, and rational defensible conclusion (take-home message). Many submitted manuscripts either lack a designated conclusion section with a relevant take-home message, or the provided conclusions are not based on the data shown in the study. The referee should assess the scientific validity of the conclusions based on the quality of the study design, appropriateness of the methodology, and scrutiny to the interpretation of the data. The conclusions must be justified by being exclusively supported by the data shown. Any speculation and hypothetical extrapolation to aspects that have not been tested in the study should be part of the discussion but not the conclusions.

### What is the overall significance of this study?

The referee should be able to identify those studies that are purely performed for the sake of publication, reflective of the classic French slogan *“l’art pour l’art”* (art for the sake of art). What are the implications of the findings and conclusions? Are the results novel and suitable for filling a gap in the existing published literature? Can the recommendations from this study potentially justify a change in surgical practice? Are the conclusions sound, and are potential shortcomings and limitations of the study addressed in the discussion of the data? Are the data clinically relevant and not just statistically significant?

## A checklist on how to write the report

- In general, the reviewer should be a steward for the submitted paper with the goal of supporting the authors’ effort by improving the final quality of a revised manuscript, whenever possible; the report should therefore be written in a positive spirit based on objective criteria and avoid any derogatory or emotional comments
- The referee should never discuss a recommendation for acceptance or rejection in the general report to authors; these comments should be reserved for the confidential comments to the editors
- We recommend to save the text of the report in regular intervals in the online submission form to avoid losing content when the website breaks down; for the same reason, the referee should always save a backup word file with the report until the review is submitted and confirmed by a feedback e-mail from the journal’s editorial system
- The report should be stratified into the following three distinct sections: general comments, major specific comments, and minor comments

### General comments

This is a short introductory section that provides a concise summary of the authors’ work in the referee’s own words. This brief synopsis of no more than 5–10 sentences should explain ‘why and how’ the study was performed (hypothesis, design, methodology) and outline a synthesis of the data with the authors’ conclusion.

### Major comments

This section is the ‘make or break’ part of the review. Some poor quality papers may indeed not pass this test and therefore be considered ‘unsalvageable’. However, most manuscripts can likely be improved secondary to the critical comments and scrutiny of the referee’s report. The major comments should be stratified according to the following considerations:

- ✓ Overall novelty and innovative aspects of the research question
- ✓ Coherence and comprehensiveness of the background section; this section should end with the specific hypothesis or stated goal of the study
- ✓ Clarity of the study hypothesis and objectives
- ✓ Adequacy of study design and methodology, including appropriate rating of the level of evidence
- ✓ The results should be presented in a logical, systematic fashion, with the presented data mirroring the same sequence as in the preceding methods section
- ✓ Soundness of statistical analysis; consider a recommendation to request an additional review by a qualified biostatistician; values of measured variables must be shown with error limits (standard deviation) and statistical significance
- ✓ Appropriateness of data interpretation and conclusions; the reported findings should be balanced relative to the context of the stated hypothesis and their scientific value placed into perspective with regards to their clinical or experimental implications
- ✓ Overall value and relevance of the study (‘So what?’ question)

### Minor comments

This section pertains to concerns of lesser importance, but still essential, which the authors should address in a revised submission:

- ✓ Clarity of writing, organization of the paper, spelling and formatting errors, inconsistent or unnecessary use of abbreviations, etc.
- ✓ The title and abstract represent the official ‘business card’ of the paper once published and available for online searches (PubMed, etc.); the referee should provide recommendations on how to improve the title and abstract, as appropriate – many working titles are too generic and not reflective of the study design and manuscript content; abstracts should represent a concise summary of the study’s main content and should ideally be structured into Background/Methods/Results/Conclusion; there should be no references cited in the abstract
- ✓ The methods section must provide a statement on study approval by the institution’s ethical review board (for clinical study) or by the animal care committee (for experimental studies), as appropriate
- ✓ Relevance, timeliness, and comprehensiveness of the cited bibliography; as a rule of thumb, about 80 % of all cited references should be representative of the peer-reviewed literature from the preceding 3–5 years
- ✓ Number and quality of figures, tables, and illustrations
- ✓ Any additional perceived concern that requires clarification, such as a potential conflict of interest by the authors (e.g. by apparent promotion of a specific surgical product instead of a surgical technique, use of company trade names instead of generic product designations, etc.), should be addressed

## Finalizing and submitting the referee’s report

The review should be written in a timely fashion and a designated referee should attempt not to miss a journal’s submission deadline – this is a sign of professional courtesy for the submitting authors and the journal’s editors who rely on the peer review system to make a decision on a submitted manuscript. After the review is completed, the referee must decide on a recommendation to the journal’s editor. In general, there is hardly ever a case of a submitted manuscript to be ‘accepted without revisions’, as the reviewer’s due diligence will always pick up some minor essential issues to help improving the quality of a revised manuscript. Submissions with only minor comments (or with a few limited major comments) should be recommended for ‘minor revisions’. In contrast, any manuscript with dramatic flaws in study design and interpretation of the data should warrant a recommendation for ‘major revisions’. Finally, selected manuscripts of extremely poor quality (or of serious ethical concern) may be deemed ‘unsalvageable’ under the presumption that the main flaws in the study design and methodology cannot be amended even if the authors are able to address all of the reviewer’s major concerns. As noted above, the confidential comments on acceptance or rejection should be exclusively addressed to the journal’s editor.

## Conclusion

A designated referee should commit to dedicating sufficient time to allow having read and understood the manuscript prior to writing any comments/critiques, and finalize the review. By following the checklist and criteria outlined in this article, any surgeon can easily become a qualified, effective, and righteous referee. We urge all future junior reviewers to go ahead and accept their refereeing invitations, to submit reports by the respective deadline, and to enjoy the reviewing assignments as an honorable and positive contribution to the scientific community and, ultimately, to our surgical patients.
